# Effects of physical fitness on mental health of Chinese college students: across-sectional study

**DOI:** 10.1186/s12889-024-18097-6

**Published:** 2024-03-06

**Authors:** Shuzhen Ma, Yang Yang, Kim Geok Soh, Hermione Tan

**Affiliations:** 1 Department of Sports Studies, Faculty of Educational Studies, Universiti Putra Malaysia, Seri Kembangan, Selangor Malaysia; 2 College of Public Administration, Guilin University of Technology, Guilin, China; 3 School of Kinesiology, Shanghai University of Sport, Shanghai, China; 4 University of the Cumberlands, Williamsburg, North America USA

**Keywords:** College students, Mental health, Physical fitness

## Abstract

**Background:**

The physical and mental health of college students is often mentioned, but there is limited research on a direct relationship between the mental health status of college students and their physical fitness level. This study mainly proves the relationship between Chinese college students’ mental health and physical fitness indicators.

**Method:**

This study collected SCL-90 Scale test results from 5262 students (4012 boys and 1250 girls) through a questionnaire survey and conducted a Sport Quality Test on these students. Statistical software SPSS was applied for differential analysis and logistic regression analysis. Specifically, the differences in sport quality indicators between normal and abnormal mean total scores of psychological tests were analyzed first. Then, the binary logistic regression model was used to explore the impacts of sports quality indicator scores on students’ psychological fitness.

**Results:**

There are differences in the results of physical fitness tests between students with abnormal psychology and students with normal psychology. The four indexes of students’ vital capacity, speed, explosive power of lower limbs, and endurance running are effective in improving students’ psychologically abnormal state, and endurance running and improving vital capacity are the most effective methods to improve students’ psychologically abnormal state. In the physical tests of Chinese college students, the risk of psychological abnormalities was reduced by 9% for every one-point increase in lung capacity and 10.4% for every one-point increase in endurance running performance.

**Conclusions:**

Chinese college students’ physical fitness and mental health are related. The best methods for treating psychological disorders are lung capacity improvement and endurance running. According to the physical test results of Chinese college students, for every 1-point increase in lung capacity and endurance running, the risk of psychological abnormalities decreased by 9% and 10.4%, respectively.

**Supplementary Information:**

The online version contains supplementary material available at 10.1186/s12889-024-18097-6.

## Background

Chinese people have tended to view college students as ‘favored by heaven’ and being less vulnerable to mental distress or disorders. With the rapid growth of universities and colleges accompanying the great socio-economic transition, however, mental health problems of college students have received unprecedented attention in recent decades in China [1, 2]. College students are prone to psychological problems due to environmental changes, academic pressure or emotional setbacks and health problems are common among both male and female college students [3]. Psychological problems will affect college students’ everyday lives, study, work, and interpersonal communication and severe cases can lead to mental illness and then the initiation of suicide. Previous mental health surveys, which mostly used self-reported dimensional scales such as the 90-Symptom Checklist (SCL-90), Self-Rating Anxiety Scale, and Self-Rating Depression Scale, have suggested a high prevalence of mental health problems (10–30%), including anxiety, phobia, obsessive and compulsive, and depression symptoms or syndromes among Chinese college students [2, 4–6]. Especially the prevalence of common mental disorders is on the rise [7–9]. The SCL-90 is a psychosomatic screening scale proposed by Derogatis that is widely used in China and elsewhere [10], and it can be used to distinguish patients with and without psychosomatic diseases and has good reliability and validity [11–13]. Chinese universities usually use SCL-90 to measure the mental health status of college students to understand their psychological status.

Based on the fact that both mental and physical aspects are considered by the World Health Organization (WHO) as integral dimensions of health and well-being [14], a strong link has been found between mental and physical health [9, 15–17], but seldom involve the pathways from one to the other [9]. Previous studies have demonstrated a close relationship between physical fitness and mental symptoms. For example, exercise improves mood [18], running can lessen loneliness and improve one’s physical well-being [19, 20], and eight weeks of swimming instruction can help college students feel better mentally [21]. However, exercise regimens in these studies must provide a comprehensive assessment of a person’s level of physical fitness. Physical fitness, commonly consisting of cardiorespiratory endurance, muscle strength endurance, flexibility, and body composition, is a vital health marker [22, 23]. There are many studies advocating the positive effects of exercise on mental health [24, 25], and most of the evidence has focused on examining the influence of moderate-to-vigorous intensity physical fitness on mental health [26]. Moderate to high-intensity sports activities are very few for Chinese students, and most students have not gone through systematic sports training. To reflect the physical health status of young people, the Ministry of Education of China issued the National Physical Health Standards for Students (revised in 2014, NPHSS), which are annually used for evaluating Chinese college students’ physical health status, thus reflecting the physical quality level of college students. The main outcomes were fitness measures and assessed by the 2014 revised Chinese National Student Physical Fitness Standard (CNSPFS), covering areas of aerobic capacity, upper body strength, flexibility, body mass index, abdominal strength, and trunk strength [23]. The national standards help to determine the goals that educators hope students will attain by the end of their educational journey [27].

In our work, we use the National Physical Health Standard for Students (NPHSS), revised in 2014, as the standard to evaluate the physical fitness level of Chinese college students and SCL-90 as a screening scale to test the mental health status of college students. The difference in average scores of sports quality index between regular and abnormal students was analyzed, and the influence of sports quality index scores on students’ psychological state was discussed using a binary logistic regression model. To provide some suggestions on how to improve the mental health status of students with abnormal mental health status in university through some physical exercises.

## Method

### Participants

This is a cross-sectional study conducted from January 2022 to January 2023 in the territory of China, including North China, Northeast China, Northwest China, and Southwest China. Eight schools were selected from each region, and each school had to select a certain number of subjects. In order to find participants, the researchers reached out to instructors of physical education and mental health instructors at eight universities. The participants willingly agreed to take the test. Students are highly motivated because they can receive credits for passing the test. According to the order of registration, each school selects the first 175 students who apply as the research object. Each region selected one thousand four hundred students from freshman to senior year. A total of 5,600 participants were tested for physical fitness and mental health, and after excluding participants with incomplete data and invalid questionnaires, data from 5,262 participating students (4,012 boys and 1,250 girls) were included in the study.

### Assessment

The study was evaluated in a physical fitness test and a questionnaire. The physical fitness test is conducted according to the test indicators in the NPHSS issued by the Ministry of Education of China. These include body mass index (BMI), lung capacity, 50 m run, sitting forward bend, standing long jump, pull-ups (men) / 1-minute sit-ups (women), 1000 m run (men) / 800 m run (women); Chinese college students are required to take a physical fitness test once a year to meet graduation requirements. The school’s physical education teacher administers the test, and all students who ask for the test have completed the physical fitness test.

Symptom Checklist-90 (SCL-90) is a widely used psychiatric questionnaire [10]. Previous studies have confirmed the good reliability of the SCL-9 scale [28]. The psychological scale was SCL-90, which consisted of 90 items. This questionnaire was divided into somatization, obsessive-compulsive, interpersonal, sensitivity, depression, anxiety, hostility, phobia, anxiety, and paranoid 10 factors of ideation and psychoticism. The teachers of eight schools sent the questionnaire to the students through mobile phones, and the students were instructed to complete the questionnaire according to the requirements. The recovery rate of the questionnaire was 100%, and the effective rate was 94%.

### Variable table

In this study, the physical quality test includes seven aspects. Due to the natural differences in physical quality between boys and girls, there are gender differences in indicator selection from Table 1. Overall, the physical quality test includes a total of seven aspects, namely: BMI, Vital capacity, 50 m, Standing long jump, Sitting forward, 1000 m (male) / 800 m (female), and Pull up (male) / One-minute sit-up (female). The connotations reflected by these seven indicators are shown in the table below.

**Table 1:** Variable table

Sport quality indicator	Indicator connotation	Corresponding variable name
BMI	BMI	BMI
Vital capacity	Vital capacity	Vital capacity
50 m	Reaction rate	Reaction rate
Standing long jump	Reflecting explosive power of lower limbs	Lower limb explosive force
Sitting forward	Reflecting flexibility	Flexibility
1000 m (male) / 800 m (female)	Reflecting endurance running	Endurance running
Pull up (male) / One-minute sit-up (female)	Reflecting muscle strength	Muscle strength

### Statistical analysis

This study collected SCL-90 Scale test results from 5262 students (4012 boys and 1250 girls) through a questionnaire survey and conducted a Sport Quality Test on these students. This study conducted frequency analysis on the total score of the sport quality test and SCL-90 scale test results of the total samples, respectively. According to gender differences, frequency analysis was also conducted for the total score of sport quality test and SCL-90 scale test results. Statistical software SPSS was used for differential analysis and logistic regression analysis. Logistic regression works similarly to linear regression but with a binomial response variable (Sperandei, 2014). Specifically, an independent sample t-test was used to compare differences between behaviors and influencing factors of energy balance among overweight/obese and normal-weight students. Logistic regression analysis was used to assess the impacts of scores of sport quality indicators on students’ psychological state. In the logistic regression analysis, the dependent variable (psychological state) is divided into two categories: normal (record as 1) and abnormal (record as 2); this study uses the binary logistic regression model to explore the impacts of scores of sports quality indicators (BMI, vital capacity, reaction rate, lower limb explosive force, flexibility, endurance running and muscle strength) on students’ psychological state.

## Results

### Total score of sport quality test

A total of 5262 students underwent physical and psychological tests in this study, including 4012 males and 1250 females. The results of the total score of the sports quality test are shown in Table 2. From Table 2, it can be seen that a total of 7 students have a total score on the sport quality test of excellent, accounting for only 0.1% of the total number; the total score of sport quality test for 289 students was good, accounting for 5.5% of the total; there are 4395 students with a total score of sport quality test of pass, accounting for 83.5% of the total number, with the highest proportion; there are 571 students with a total score of sport quality test of failure, accounting for 10.9% of the total number. Among boys, 0.1% were rated excellent, 5.5% good, 83.5% passed, and 10.9% failed. The proportion of excellent was 0.3%, the proportion of excellent was 5.7%, the proportion of qualified was 91.4%, and the proportion of unqualified was 2.6%. The proportion of girls who test as excellent, good, and pass is higher than that of boys, and the proportion of boys who test as failing is higher than that of girls.

**Table 2:** Frequency table of total score of sport quality test

Total score of sport quality test	Overall	Boys	Girls
Excellent	7 (0.1%)	3 (0.1%)	4 (0.3%)
Good	289 (5.5%)	218 (5.4%)	71 (5.7%)
Pass	4395 (83.5%)	3253 (81.1%)	1142 (91.4%)
Failure	571 (10.9%)	538 (13.4%)	33 (2.6%)
Total	5262 (100%)	4012 (100%)	1250 (100%)

### The SCL-90 scale test results

Table 3 shows the frequency distribution of SCL-90 scale test results. From Table 3, in terms of the ten dimensions of SCL-90 Scale scores, the number of students with a psychological test result of normal is higher than that with an abnormal result, and the number of students with a psychological test result of abnormal accounts for a relatively low proportion of the total number. Among the normal indicators, 95.4% were somatization, 80.0% obsessive-compulsive disorder, 86.6% interpersonal sensitivity, 89.3% depression, 91.9% anxiety, 92.6% hostility, 93.6% terror, 92% paranoia, and 92.2% psychoticism. Others accounted for 90.4%. The average total score 92.7%. For girls, 94.6% of the indicators tested as normal were somatization, 76% obsessive-compulsive disorder, 84.6% interpersonal sensitivity, 85.8% depression, 90.3% anxiety, 91% hostility, 92.2% terror, 91.7% paranoia, and 92.4% psychoticism. Others accounted for 88.6%, and the average total score 91.8%. The proportion of normal in somatization, obsessive-compulsive disorder, interpersonal sensitivity, depression, anxiety, hostility, terror, paranoia, and other aspects and total score are all higher in boys than girls, and the proportion of normal in girls is higher than boys only in psychoticism.

**Table 3:** Frequency table of SCL-90 scale test results

SCL-90 scale test results		Overall		Boys		Girls	
		Frequency	Percent (%)	Frequency	Percent (%)	Frequency	Percent (%)
Somatization	Normal	5016	95.3	3834	95.6	1182	94.6
	Abnormal	246	4.7	178	4.4	68	5.4
Obsessive-compulsive	Normal	4211	80.0	3261	81.3	950	76.0
	Abnormal	1051	20.0	751	18.7	300	24.0
Interpersonal sensitivity	Normal	4533	86.1	3475	86.6	1058	84.6
	Abnormal	729	13.9	537	13.4	192	15.4
Depression	Normal	4700	89.3	3628	90.4	1072	85.8
	Abnormal	562	10.7	384	9.6	178	14.2
Anxiety	Normal	4834	91.9	3705	92.3	1129	90.3
	Abnormal	428	8.1	307	7.7	121	9.7
Hostility	Normal	4873	92.6	3735	93.1	1138	91.0
	Abnormal	389	7.4	277	6.9	112	9.0
Phobic anxiety	Normal	4926	93.6	3774	94.1	1152	92.2
	Abnormal	336	6.4	238	5.9	98	7.8
Paranoid ideation	Normal	4842	92.0	3696	92.1	1146	91.7
	Abnormal	420	8.0	316	7.9	104	8.3
Psychoticism	Normal	4849	92.2	3694	92.1	1155	92.4
	Abnormal	413	7.8	318	7.9	95	7.6
Others	Normal	4758	90.4	3650	91.0	1108	88.6
	Abnormal	504	9.6	362	9.0	142	11.4
Mean total score	Normal	4877	92.7	3730	93.0	1147	91.8
	Abnormal	385	7.3	282	7.0	103	8.2
Total		5262	100.0	4012	100.0	1250	100.0

### Differences of sport quality indicators between normal and abnormal mean total score of psychological test (*N* = 5262)

Table 4 displays the differences in sport quality indicators between normal and abnormal mean total scores of psychological tests. In the overall sample, no differences in sport quality indicators of BMI, lower limb explosive force, flexibility, and muscle strength were observed between the two groups (*p* > 0.05); however, the scores of vital capacities, reaction rate, and endurance running were significantly lower in the abnormal psychology state than in the normal psychology state (*p* < 0.01). In the male sample, no differences in sport quality indicators of BMI, reaction rate, lower limb explosive force, flexibility, and endurance running were observed between the two groups (*p* > 0.05). However, the vital capacities, muscle strength, and endurance running scores were significantly lower in abnormal psychology than in normal psychology (*p* < 0.05). In the female sample, no differences in sport quality indicators of BMI, lower limb explosive force, flexibility, and muscle strength were observed between the two groups (*p* > 0.05). However, vital capacity, reaction rate, and endurance running were significantly lower in abnormal psychology than in normal psychology (*p* < 0.01).

**Table 4:** Differences of sport quality indicators between normal and abnormal mean total score of psychological test (*N* = 5262)

	Overall (*n* = 5262)				Boys (*n* = 4012)				Girls (*n* = 1250)			
	Normal Psychology State	Abnormal Psychology State	*p* value	95% CI	Normal Psychology State	Abnormal Psychology State	*p* value	95% CI	Normal Psychology State	Abnormal Psychology State	*p* value	95% CI
BMI	94.52 ± 9.92	94.18 ± 10.07	0.523	(-0.695, 1.366)	93.75 ± 10.42	93.62 ± 10.49	0.839	(-1.131, 1.393)	97.02 ± 7.55	95.73 ± 8.70	0.148	(-0.464, 3.045)
Vital capacity	74.69 ± 10.30	62.93 ± 19.44^a^	0.000	(9.790, 13.729)	74.36 ± 9.56	64.10 ± 18.20 ^a^	0.000	(8.109, 12.420)	75.76 ± 12.34	59.74 ± 22.28 ^a^	0.000	(11.610, 20.434)
Reaction rate	70.18 ± 4.87	67.99 ± 9.24 ^a^	0.000	(1.253, 3.124)	71.22 ± 4.59	70.48 ± 5.39	0.010	(0.180, 1.306)	66.80 ± 4.17	61.18 ± 13.31 ^a^	0.000	(3.001, 8.225)
Lower limb explosive force	62.76 ± 17.96	61.71 ± 18.21	0.269	(-0.813, 2.919)	61.33 ± 18.51	59.98 ± 18.88	0.238	(-0.893, 3.595)	67.43 ± 15.15	66.46 ± 15.36	0.532	(-2.086, 4.034)
Flexibility	72.97 ± 16.12	72.92 ± 16.67	0.955	(-1.629, 1.726)	71.39 ± 16.23	70.80 ± 17.16	0.558	(-1.384, 2.563)	78.07 ± 14.64	78.70 ± 13.75	0.677	(-3.564, 2.314)
Endurance Running	72.57 ± 6.96	65.44 ± 14.49 ^a^	0.000	(5.662, 8.593)	72.09 ± 6.80	65.59 ± 13.69	0.000	(4.886, 8.124)	74.14 ± 7.24	65.06 ± 16.57	0.000	(5.813, 12.344)
Muscle strength	41.39 ± 31.79	41.95 ± 31.75	0.739	(-3.859, 2.739)	34.04 ± 32.50	33.78 ± 32.68	0.898	(-3.679, 4.194)	65.29 ± 10.80	64.31 ± 12.80	0.385	(-1.234, 3.195)

^a^ means there is significant difference at 0.01 level

### Logistic regression analysis

The binary logistic regression model is applied to explore the impacts of scores of sport quality indicators on students’ psychological state because the dependent variable (psychological state) is divided into two categories: normal (record as 1) and abnormal (record as 2). The results are shown in Table 5. In the overall sample, the regression coefficients of the independent variables, vital capacity (*p* < 0.01), rate (*p* < 0.01), lower limb exploratory force (*p* < 0.05), and endurance running (*p* < 0.01) were significant, while the regression coefficients of the other three independent variables BMI (*p* > 0.05), flexibility (*p* > 0.05), and muscle strength (*p* > 0.05) were not significant. From this, it can be concluded that four factors affect students’ psychological state in the overall samples, namely, vital capacity, reaction rate, lower limb exploratory force, and endurance running. Furthermore, it can be seen that for every 1-point increase in vital capacity score, the risk of students’ psychological state being abnormal will decrease by 9%; when the rating score increases by 1-point, the risk of a student’s psychological state being abnormal decreases by 2.1%; when the lower limb exploratory force score increases by1-point, the risk of a student’s psychological state being abnormal increases by 0.9%; when the endurance running score increases by 1 point, the risk of a student’s psychological state being abnormal decreases by 10.4%.

**Table 5:** Results of Sport Quality Indicators Logistic Regression Analysis Between Normal and Abnormal Mean Total Score of Psychological Test (*N* = 5262)

	Overall (*n* = 5262)				Boys (*n* = 4012)				Girls (*n* = 1250)			
	B	*p*	OR	95% CI	B	*p*	OR	95% CI	B	*p*	OR	95% CI
BMI	0.004	0.561	1.004	(0.992, 1.016)	0.008	0.241	1.008	(0.995, 1.022)	-0.027	0.054	0.973	(0.947, 1.001)
Vital capacity	-0.090	0.000	0.914	(0.903, 0.926)	-0.087	0.000	0.917	(0.904, 0.930)	-0.100	0.000	0.905	(0.881, 0.929)
Reaction rate	-0.041	0.000	0.960	(0.938, 0.982)	0.004	0.846	1.004	(0.967, 1.042)	-0.141	0.000	0.868	(0.819, 0.920)
·Explosive force	0.009	0.021	1.009	(1.001, 1.016)	0.003	0.498	1.003	(0.994, 1.012)	0.027	0.014	1.027	(1.006, 1.050)
Flexibility	0.000	0.903	1.000	(0.992, 1.007)	0.000	0.991	1.000	(0.992, 1.008)	0.005	0.569	1.005	(0.987, 1.025)
Endurance Running	-0.104	0.000	0.901	(0.887, 0.916)	-0.114	0.000	0.892	(0.874, 0.911)	-0.085	0.000	0.919	(0.892, 0.946)
Muscle strength	0.003	0.212	1.003	(0.999, 1.007)	0.003	0.160	1.003	(0.999, 1.008)	0.004	0.754	1.004	(0.982, 1.026)
Constant	12.849	0.000	380495.610	-	10.086	0.000	24004.665	-	19.801	0.000	397613454.719	-

B represents the non-standardized coefficient, P represents the significance, OR represents the odds ratio and 95% CI represents the 95% confidence interval

In the male sample, the regression coefficients of the independent variables vital capacity (*p* < 0.01) and endurance running (*p* < 0.01) were significant, while the regression coefficients of the other five independent variables BMI (*p* > 0.05), action rate (*p* > 0.05), lower limb exploratory force (*p* > 0.05), flexibility (*p* > 0.05), and muscle strength (*p* > 0.05) were not significant. From this, two factors affect male students’ psychological state: vital capacity and endurance running. From Table 5, it can be further inferred that for every 1-point increase in vital capacity score, the risk of male students having an abnormal psychological state decreases by 8.7%; when the endurance running score increases by 1 point, the risk of male students experiencing an abnormal psychological state decrease by 11.4%.

In the female sample, the regression coefficients of the independent variables vital capacity (*p* < 0.01), action rate (*p* < 0.01), lower limb exploratory force (*p* < 0.05), and endurance running (*p* < 0.01) were significant, while the regression coefficients of the other three independent variables BMI (*p* > 0.05), flexibility (*p* > 0.05), and muscle strength (*p* > 0.05) were not significant. From this, it can be concluded that four factors affect the psychological state of female students in the female sample, namely, vital capacity, reaction rate, lower limb exploratory force, and endurance running. From Table 5, it can be further seen that for every 1-point increase in vital capacity score, the risk of girls’ psychological state being abnormal will decrease by 10%; when the rate score increases by 1 point, the risk of a girl’s psychological state being abnormal decreases by 14.1%; when the lower limb exploratory force score increases by 1 point, the risk of girls having an abnormal psychological state increases by 2.7%; when the endurance running score increases by 1 point, the risk of a girl’s psychological state being abnormal decreases by 8.5%.

## Discussion

This study collected the test results of the SCL-90 scale from 5262 students (4012 boys and 1250 girls) through a questionnaire survey. Previous studies have confirmed the good reliability of the SCL-9 scale [29]. In this study, the SCL-90 scale test results show that students with normal psychological indicators are far more than those with abnormal psychological indicators. In terms of the proportion of male and female test results, the proportion of somatization, obsessive-compulsive disorder, interpersonal sensitivity, depression, anxiety, hostility, terror, paranoia, and normal total scores are all higher in male students than in female students. In comparison, the proportion of normal in female students is higher than that in male students only in psychiatric disorders. These problems may escape their parents’ attention, leading to problems later in life [30].

In this study, the psychological survey of these students was carried out simultaneously with the sports quality test. Through the sports quality test, we can see that the test results of Chinese college students accounted for the largest proportion of passing, failing more, good closely followed, and excellent tests very few. This finding relates to China’s many inactive people, accounting for two-thirds of the total population [30]. Especially during the COVID-19 outbreak, the prevalence of insufficient physical fitness among Chinese citizens is more than double the global level, and the time spent watching screens is more than four hours a day, with young people spending the most time watching screens [31]. This finding is also why only some excellent college students take the exercise test, and many fail it.

Through the analysis of the difference in the average total score of psychological tests between normal and abnormal subjects, the scores of vital capacity, speed, and endurance running were significantly lower than those of normal psychological state. The scores of vital capacity and muscle strength in male samples were significantly lower than those in normal psychological states, and the scores of vital capacity, speed, and endurance running in female samples were significantly lower than those in normal psychological states. This finding shows that the test subjects with normal mental states are higher than the test subjects with abnormal mental states in some sports abilities, and participating in sports and exercise is beneficial to mental state and can improve mood [32]. The indicators affecting male and female subjects differ slightly. A study that looks at gender variations in exercise motivation and behavior from the standpoint of evolutionary psychology explains this. According to the study, Men’s motivation for exercise is centered on building muscle mass and improving upper body contour, whereas women’s motivation is centered on weight loss and lower body enhancement [33]. Men and women have distinct motivations, resulting in distinct sources of happiness.

Based on the Binary Logistic Regression Model, it was found that there are four factors affecting students’ psychological state in the whole sample, namely, vital capacity, reaction rate, lower limb exploration force, and endurance running. Among these four indicators, speed and lower limb explosive power have a specific effect on reducing psychological abnormalities, which is mainly manifested in girls. However, the most effective indicators are endurance running and lung capacity, which positively affect both boys and girls. While numerous studies recently support the beneficial effects of exercise on mental health [34–40]. However, there is no consensus on which type of exercise is superior as both aerobic and anaerobic exercise can enhance mental health [41–43], and our findings unmistakably demonstrate that aerobic exercise is more efficient compared to anaerobic exercise. There is a strong correlation between Lung capacity and endurance running, and running for long periods is one way to increase lung capacity. Numerous earlier studies have indicated that endurance running is beneficial for mental health. In an early study by Vezina et al., consistent running lowers anxiety, boosts self-esteem, and produces positive mood shifts [44]. Research on marathon training revealed a favorable correlation between the sport and psychological coping and self-worth [45, 46]. According to Kerr’s study, university students who regularly exercised for seven weeks without any controls and who ran weekly 40-minute fixed-distance outdoor rural runs reported happier moods. Those who ran faster scored higher than those who ran slower [47]. Few research studies have been done on the connection between mental health and exercise types other than endurance running, but it is a topic worth looking into in the future.

Maintaining physical fitness can improve mental health through the common inflammatory and neurochemical pathways; the neurochemical effects of physical fitness reduce the risk of the onset of particular mental disorders [48–50]. On the other hand, the opposite explanation (i.e., having a mental disorder may be a barrier to physical fitness) also accounts for the observed impacts of physical fitness on mental health [51]. For instance, a prior study found that individuals with social phobia are less likely to play team sports or engage in active recreational activities with others out of fear of being negatively judged by others [52]. It is also possible that those who experience mental health symptoms have lower energy or are more apathetic, which makes them less likely to exercise [53]. On the other hand, mental well-being might also raise the likelihood of engaging in physical fitness [54].

This study shows that exercise effectively brings about positive mental health changes in Chinese college students [55]. Moreover, it has been proved that the most effective way to improve psychological abnormalities is to conduct endurance running and improve lung capacity. Our future research will build on this investigation and continue to explore the effects of different types of intensity physical fitness on mental health.

## Conclusions

This study found that exercise is effective in bringing about positive changes in Chinese college students’ mental health, and endurance running and improving lung capacity are the most effective ways to improve mental abnormalities. In the physical test results of Chinese college students, the risk of psychological abnormalities decreased by 9% for every 1-point increase in lung capacity, and the risk of psychological abnormalities decreased by 10.4% for every 1-point increase in endurance running.

### Electronic supplementary material

Below is the link to the electronic supplementary material.

Supplementary Material 1

## Data Availability

The datasets used and/or analysed during the current study are available from the corresponding author on reasonable request. If there is any need, please contact the author (Shuzhen Ma - email address: msz20210607@126.com).

## Abbreviations

SCL: Symptom Checklist
WHO: World Health Organization
NPHSS: National Physical Health Standards for Students
CNSPFS: Chinese National Student Physical Fitness Standard
BMI: Body mass index
