# Supplementary material for: Effects of physical fitness on mental health of Chinese college students: across-sectional study
Source: BMC Public Health. 2024 Mar 6;24:727. doi: 10.1186/s12889-024-18097-6 (PMC10918864; doi:10.1186/s12889-024-18097-6)
Supplement: Supplementary file 1 — Supplementary Material 1 [file 12889_2024_18097_MOESM1_ESM.pdf]

Name__________________________ ID# ____________________Date _______________

SCL-90 Symptom Self-Assessment scale

Below is a list of problems and complaints that people sometimes have. Please read each one carefully. After you have done so, select one of the numbered descriptors that best describes HOW MUCH THAT PROBLEM HAS BOTHERED OR DISTRESSED YOU DURING THE PAST WEEK, INCLUDING TODAY. Circle the number in the space to the right of the problem and do not skip any items. Use the following key to guide how you respond:

Circle 0 if your answer is NOT AT ALL
Circle 1 if A LITTLE BIT
Circle 2 if MODERATELY
Circle 3 if QUITE A BIT
Circle 4 if EXTREMELY

Please read the following example before beginning:

Example: In the previous week, how much were you bothered by:

Backaches 0 (1) 2 3 4

In this case, the respondent experienced backaches a little bit (1).
Please proceed with the questionnaire.

	HOW MUCH WERE YOU BOTHERED BY:	NOT AT ALL	A LITTLE BIT	MODERATELY	QUITE A BIT	EXTREMELY
1.	Headaches	0	1	2	3	4
2.	Nervousness or shakiness inside	0	1	2	3	4
3.	Unwanted thoughts, words, or ideas that won't leave your mind	0	1	2	3	4
4.	Faintness or dizziness	0	1	2	3	4
5.	Loss of sexual interest or pleasure	0	1	2	3	4
6.	Feeling critical of others	0	1	2	3	4
7.	The idea that someone else can control your thoughts	0	1	2	3	4
8.	Feeling others are to blame for most of your troubles	0	1	2	3	4
9.	Trouble remembering things	0	1	2	3	4
10.	Worried about sloppiness or carelessness	0	1	2	3	4
11.	Feeling easily annoyed or irritated	0	1	2	3	4
12.	Pains in heart or chest	0	1	2	3	4
13.	Feeling afraid in open spaces or on the streets	0	1	2	3	4
14.	Feeling low in energy or slowed down	0	1	2	3	4
15.	Thoughts of ending your life	0	1	2	3	4
16.	Hearing voices that other people do not hear	0	1	2	3	4
17.	Trembling	0	1	2	3	4
18.	Feeling that most people cannot be trusted	0	1	2	3	4
19.	Poor appetite	0	1	2	3	4

SCL-90 (*continued*)

	HOW MUCH WERE YOU BOTHERED BY:	NOT AT ALL	A LITTLE BIT	MODERATELY	QUITE A BIT	EXTREMELY
20.	Crying easily	0	1	2	3	4
21.	Feeling shy or uneasy with the opposite sex	0	1	2	3	4
22.	Feeling of being trapped or caught	0	1	2	3	4
23.	Suddenly scared for no reason	0	1	2	3	4
24.	Temper outbursts that you could not control	0	1	2	3	4
25.	Feeling afraid to go out of your house alone	0	1	2	3	4
26.	Blaming yourself for things	0	1	2	3	4
27.	Pains in lower back	0	1	2	3	4
28.	Feeling blocked in getting things done	0	1	2	3	4
29.	Feeling lonely	0	1	2	3	4
30.	Feeling blue	0	1	2	3	4
31.	Worrying too much about things	0	1	2	3	4
32.	Feeling no interest in things	0	1	2	3	4
33.	Feeling fearful	0	1	2	3	4
34.	Your feelings being easily hurt	0	1	2	3	4
35.	Other people being aware of your private thoughts	0	1	2	3	4
36.	Feeling others do not understand you or are unsympathetic	0	1	2	3	4
37.	Feeling that people are unfriendly or dislike you	0	1	2	3	4
38.	Having to do things very slowly to insure correctness	0	1	2	3	4
39.	Heart pounding or racing	0	1	2	3	4
40.	Nausea or upset stomach	0	1	2	3	4
41.	Feeling inferior to others	0	1	2	3	4
42.	Soreness of your muscles	0	1	2	3	4
43.	Feeling that you are watched or talked about by others	0	1	2	3	4
44.	Trouble falling asleep	0	1	2	3	4
45.	Having to check and double-check what you do	0	1	2	3	4
46.	Difficulty making decisions	0	1	2	3	4
47.	Feeling afraid to travel on buses, subways, trains	0	1	2	3	4
48.	Trouble getting your breath	0	1	2	3	4
49.	Hot or cold spells	0	1	2	3	4
50.	Having to avoid certain things, places, or activities because they frighten you	0	1	2	3	4
51.	Your mind going blank	0	1	2	3	4
52.	Numbness or tingling in parts of your body	0	1	2	3	4
53.	A lump in your throat	0	1	2	3	4
54.	Feeling hopeless about the future	0	1	2	3	4
55.	Trouble concentrating	0	1	2	3	4

SCL-90 (*continued*)

HOW MUCH WERE YOU BOTHERED BY:		NOT AT ALL	A LITTLE BIT	MODERATELY	QUITE A BIT	EXTREMELY
56.	Feeling weak in parts of your body	0	1	2	3	4
57.	Feeling tense or keyed up	0	1	2	3	4
58.	Heavy feelings in your arms or legs	0	1	2	3	4
59.	Thoughts of death or dying	0	1	2	3	4
60.	Overeating	0	1	2	3	4
61.	Feeling uneasy when people are watching or talking about you	0	1	2	3	4
62.	Having thoughts that are not your own	0	1	2	3	4
63.	Having urges to beat, injure, or harm someone	0	1	2	3	4
64.	Awakening in the early morning	0	1	2	3	4
65.	Having to repeat the same actions such as touching, counting, washing	0	1	2	3	4
66.	Sleep that is restless or disturbed	0	1	2	3	4
67.	Having urges to break or smash things	0	1	2	3	4
68.	Having ideas or beliefs that others do not share	0	1	2	3	4
69.	Feeling very self-conscious with others	0	1	2	3	4
70.	Feeling uneasy in crowds, such as shopping or at a movie	0	1	2	3	4
71.	Feeling everything is an effort	0	1	2	3	4
72.	Spells of terror or panic	0	1	2	3	4
73.	Feeling uncomfortable about eating or drinking in public	0	1	2	3	4
74.	Getting into frequent arguments	0	1	2	3	4
75.	Feeling nervous when you are left alone	0	1	2	3	4
76.	Others not giving you proper credit for your achievements	0	1	2	3	4
77.	Feeling lonely even when you are with people	0	1	2	3	4
78.	Feeling so restless you couldn’t sit still	0	1	2	3	4
79.	Feelings of worthlessness	0	1	2	3	4
80.	Feeling that familiar things are strange or unreal	0	1	2	3	4
81.	Shouting or throwing things	0	1	2	3	4
82.	Feeling afraid you will faint in public	0	1	2	3	4
83.	Feeling that people will take advantage of you if you let them	0	1	2	3	4
84.	Having thoughts about sex that bother you a lot	0	1	2	3	4
85.	The idea that you should be punished for your sins	0	1	2	3	4
86.	Feeling pushed to get things done	0	1	2	3	4
87.	The idea that something serious is wrong with your body	0	1	2	3	4
88.	Never feeling close to another person	0	1	2	3	4
89.	Feelings of guilt	0	1	2	3	4
90.	The idea that something is wrong with your mind	0	1	2	3	4
